# Statistics of the Globe

**Published:** 1863-04

**Authors:** 


### STATISTICS OF THE GLOBE.

The following curious facts are stated by the *Abeille Medicale:*—The earth is inhabited by about 1,288,000,000 of inhabitants, viz: 369,000,000 of the Caucasian race, 552,000,000 of the Mongolian race, 196,000,000 of the American Indians, and 200,000,000 of the Malay races. All these respectively speak 3064 languages, and profess 1000 different religions.—The amount of deaths per annum is 333,333,333, or 91,954 per day, 3730 per hour, 60 per minute, or 1 per second; so that at every pulsation of our hearts, a human being dies. This loss is compensated by an equal number of births. The average duration of life throughout the globe is 33 years. One-fourth of this population dies before the seventh year, and one-half before the seventeenth. Out of 10,000 persons only one reaches his hundredth year, only one in 500 his eightieth, and only one in 100 his sixty-fifth. Married people live longer than unmarried ones, and a tall man is likely to live longer than a short one. Until the fiftieth year women have a better chance of life than men, but beyond that period the chances are equal. Sixty-five persons out of one thousand marry. The months of June and December are those in which marriages are most frequent.—Children born in spring are generally stronger than those born in other seasons. Births and deaths chiefly occur at night. The number of men able to bear arms is but an eighth of the population. The nature of the profession exercises a great influence on longevity; thus out of 100 of each of the following professions the number of those who attain their seventieth year is—among clergymen, 42; agriculturalists, 40; trades and manufacturers, 33; soldiers, 32; clerks, 32; lawyers, 29; artists, 28; professors, 27; and physicians, 24; so that those who study the art of prolonging the lives of others are most liable to die early, probably on account of the effluvia to which they are constantly exposed. There are in the world 335,000,000 of Christians, 5,000,000 of Jews, 600,000,000 professing some of the Asiatic religions, 160,000,000 of Mahometans, and 200,000,000 of Pagans. Of the Christians, 170,000,000 profess the Roman Catholic, 76,000,000 the Greek, and 80,000,000 the Protestant creeds.—*Boston Medical Journal.*

